# Practical Aspects of Cardiopulmonary Exercise Testing in Children

**DOI:** 10.1055/a-2191-0518

**Published:** 2023-11-29

**Authors:** Tim Takken, Erik H. Hulzebos

**Affiliations:** 1 Child Development & Exercise Center, Wilhelmina Children’s Hospital, UMC Utrecht – Locatie WKZ, Utrecht, Netherlands

**Keywords:** physiology, child, exercise test, fitness

## Abstract

The use of cardiopulmonary exercise testing (CPET) in pediatrics provides critical insights into potential physiological causes of unexplained exercise-related complaints or symptoms, as well as specific pathophysiological patterns based on physiological responses or abnormalities. Furthermore, CPET helps evaluate exercise performance in children with chronic (lung/heart) diseases. For instance, it can ascertain any adverse reaction to exercise and estimate the effects of specific treatment measures. It affords a global assessment of the pathophysiological patterns, responses and abnormalities to exercise that is inadequately reflected by resting lung function and/or cardiac function assessment. Clinical interpretation of the results of a CPET in pediatrics requires specific knowledge regarding pathophysiological responses and interpretative strategies that can be adapted to address concerns specific to the child’s medical condition or disability.

## Introduction

Cardiopulmonary exercise testing (CPET) is a commonly used procedure to assess the physiological response to exercise in subjects. Aerobic fitness is currently seen as a vital sign in the adult population 1 , as well as in the pediatric age group 2 . However, most clinicians lack education, expertise, experience, usage and successes applying the benefits of CPET for their pediatric patients, because it is relatively new and not widely taught. There are many perceived barriers to using CPET in clinical practice. Many pediatricians do not know how to perform a CPET, and/or what to do with the test results, or do not have the equipment to perform a CPET. This means that CPET is still quite underused in pediatrics. In this article, we will provide some practical advice on performing CPET in children and selecting reference values, and we will outline the approaches to CPET performance and interpretation that proved more helpful in managing cardio-respiratory patients.

## Why is CPET employed in children?

There are several indications for performing CPET in children and adolescents 3 . First, CPET can be employed as a diagnostic test, for example, to assess aerobic fitness or to examine abnormal exercise responses (exercise-induced dyspnea, exercise-induced tachycardia, exercise-related syncope, etc.).

Second, CPET can be used for the assessment of disease severity, for example, for patients with (congenital) heart disease or lung disease (hypoxia, gas exchange abnormalities, dysfunctional breathing, etc.). Although myocardial ischemia is a very rare adverse event during CPET in children, in contrast to CPET in (older) adults, CPET is often done to rule out cardiac ischemia in children with chest pain. The very low occurrence of myocardial ischemia is a major difference in CPET between children and adults.

Third, CPET can be used as a prognostic test. For example, CPET is used as a tool for screening whether a patient is at risk for future cardiac-related events 4 .

Fourth, CPET can be used as an evaluative test, for example, to test the effectiveness of an intervention program such as exercise training 5 . CPET can also be used in the regular follow-up for patients with a progressive disease such as cystic fibrosis 6 .

Fifth, CPET can assist in generating a personalized exercise prescription for children with an acute or chronic disease.

Sixth, CPET and pediatric biometric testing is invaluable for all our acute/chronic disease pediatric patients for baseline appraisal, tracking progress, and most importantly to encourage regular daily exercise patterns.

## How to do CPET in children?

The main principles for CPET in children are comparable to the testing of adults. An ergometer is used to increase the workload until volitional fatigue of the subject. The testing room should have sufficient ventilation and be child-friendly. Also, the staff should have experience with working with children. CPETs are often performed by two operators. The pediatric testing is often performed by PhD/MD level exercise physiologists together with a MSc-level technician/exercise physiologist. In the following section, equipment and protocols will be described.

## Materials and Methods

### Equipment

The equipment should be appropriate for use in children. For example, the face mask should be small enough to fit the child’s face without leaks. There is a variety of masks available ranging from “pediatric large” to ”adult large”. The oxygen saturation probe should fit the child’s finger, earlobe or forehead. Usually there is a sensor for children<30 kg of body mass, and a sensor for over 30 kg of body mass.

The blood pressure cuff should be small enough to fit the child’s arm. The flow sensor should be sensitive enough to measure the low airflows observed in small children. We advise performing a flow calibration for low air flows when a CPET is performed in a child.

Also, the ergometer used should have the correct configuration for the child (handrail height of treadmill, saddle height, reach to the handlebar, as well as the width and the length of the crankset). Usually, a child should be at least 125 cm tall to fit commonly used cycle ergometers; this is somewhat dependent on ergometer type, saddle, and crank length. We test children smaller than 125 cm on a special pediatric cycle ergometer with short cranks and a small handlebar and saddle.

### Protocols for workload increase

The subject must exercise against an incremental load until exhaustion. It is important that the exercise is done with large muscle groups (>50% of the muscle mass is involved). Therefore, the main mode of exercise testing is with the lower extremities (e. g. walking/running or cycling). Pediatric patients typically take 90–120 seconds to adapt a level of homeostasis at each workload level. So, if steady state values are important to record during exercise, a stepwise protocol with 1.5 to 3 min steps is appropriate to use. When steady-state values are less important, a ramp wise protocol can be used.

Also, for testing children, many different protocols for the increase in workload are available. Each protocol comes with its pros and cons. Important considerations for choosing a protocol are listed in Table 1 .

**Table:** **Table 1** Considerations for choosing a CPET protocol.

*Feature*	*Consideration*
*Starting speed*	*If starting speed is too high, subjects will terminate a test very rapidly*
*Starting incline*	*If starting incline is too high, subjects will stop because of musculoskeletal discomfort instead of cardiopulmonary capacity.*
*Stage duration*	*A short stage duration<1 min will give a smoother gas-exchange response and will help to identify the ventilatory anaerobic threshold (VAT/VT1) and the respiratory compensation point (RCP/VT2)*
*Exercise mode*	*The main modes are walking/running or cycling. Walking/running has the advantage of resulting in a somewhat higher VO*_*2max*_ *compared to cycling. Also there is no size limit for running, while many cycle ergometers need at least a subjects’ height of 125 cm. The cycle ergometer has the advantage of resulting in more stable measurements of the exercise ECG. Also if blood samples are required, cycle ergometry is preferred.*
*Reference values available*	*Some exercise parameters are dependent on the exercise mode and/or protocol. Therefore, one should choose a CPET protocol for which reference values are developed, when the indication for the CPET is employed as a diagnostic test.*
*Protocol used in comparable patient population?*	*Some patient populations require a special protocol since standard protocols like the Bruce test is not suited for testing the patient. For example, special protocols are developed for pediatric patients with spina bifida, or cerebral palsy.*

One of the major advantages of using the cycle ergometer and not the treadmill for CPET is the fact that the accuracy and precision of the workload and power output is best controlled on the cycle ergometer. Furthermore, many measurements (e. g. blood pressure, ECG) are easier to perform while exercising on a stationary cycle ergometer than running on a treadmill.

### Cycle ergometry protocols

For cycle ergometry, the Godfrey protocol is often used. This protocol is based on height: for<125 cm, the workload is increased by 10 Watt/min, for 125–150 cm, by 15 Watt/min, and for>150 cm by 20 Watt/min increase 7 .

Another approach is an individualized protocol as described by Karila et al. 8 . Individualization of the workload protocol for cycle ergometry helps to perform a CPET within the recommended duration of 6–10 or 8–12 minutes. This estimate, based on the predicted VO _2peak_ of each child, converted into a maximum workload (W _peak_ ), makes it easier to set the workload increment during the test. This will result in an individualized cycle protocol unique to each patient.

This can be done as follows (After 8 ):

Calculate basal VO _2_ =(height in centimeters x 2) – 100.(1)

**Fig. 1:**
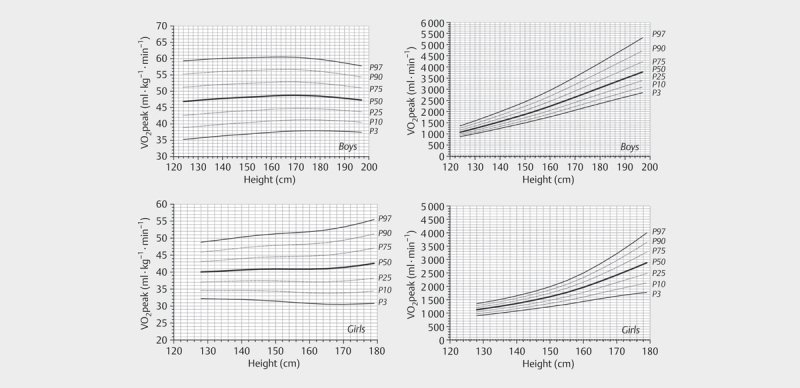
The VO _2peak_ and VO _2peak_ per kilogram body mass in relation to height for boys and girls. Data reanalyzed after 3 with permission.

Calculate predicted VO _2peak_ from Fig. 1 .(2)

Calculate predicted W _peak_ =(predicted VO _2peak_ – basal VO _2_ )/10.3(3)

where 10.3 mL of O _2_ /min/W is the equivalent in oxygen of each watt.

The total duration of the test should be between 8 to 12 min. The test involves four consecutive periods: (1) a 3-min to 5-min rest period; (2) a 3-min period of warm-up against an unloaded workload; and (3) an 8–12-min incremental exercise period. The minute steps (preferably programmed as a linear RAMP-wise protocol) of the W _peak_ can be divided by 10 to define the increase in workload for each 1-min stage.

A recovery period (4), with a workload equivalent to that used for the 1-min increment of at least 2 min, is necessary to prevent fainting and to accelerate recovery. We recommend having a stable cadence of 60 to 80 RPM during cycling. Finally, there are 3 min of passive recovery. This individualized methodology should make it possible to expand the use of CPET in pediatrics, both for diagnosis and treatment.

### Treadmill protocols

There are many protocols available for treadmill testing. Well-known protocols are the Bruce 9 , Fitkids 10 and the Dubowy protocol 11 , among others. All protocols increase the workload using a combination of time, speed and incline. The Bruce protocol was originally developed for testing adult cardiac patients. Drawbacks include the high incline of the treadmill, the uneven steps in speed, and the relatively large step duration (3 minutes). Therefore, many modifications have been made to the original Bruce protocol. For patients with a low exercise capacity one should use a protocol with very low-low treadmill exertions per incline, speed & incremental effort.

The Fitkids treadmill test was developed to test 6 to 18-year-old children with chronic medical diseases. With an initial low incline and speed of the protocol, it is also suitable for testing patients with a low cardiorespiratory fitness (CRF). The protocol can be used in patients with different medical conditions 12 . Reference values were also developed for this age group 13 .

The Dubowy protocol was developed for use in a wide age group (4–75 years) 11 for the long-term follow-up of congenital heart disease patients. Reference values were also developed for this age range, making this protocol very attractive to use in follow-up because no change in reference values is needed during the transition into adulthood.

### Encouragements

We cannot stress enough the importance of encouragement during the CPET. Strong verbal encouragement is important to bring the child to its peak effort. Currently, there are no standardized operation procedures for encouragement available for pediatric CPET, as are used in walk tests. When no encouragement is given, many children will give a sub-maximal effort, which limits the interpretability of the CPET data.

In addition, the child’s Rating of Perceived Exertion (RPE) can be assessed every 60 seconds on a 0–10 or 6–20 scale 14 to track the perceived effort. The goal is 9/10 or 18/20 at peak exercise on the two RPE scales respectively. In addition, RPE for the legs can be asked, since muscle function is a major limiting factor in pediatric CPET.

### Influence of growth and development

The most important functions of the cardiopulmonary system during exercise are to deliver oxygen and nutrients to the exercising muscles and heart, and to remove the metabolically produced carbon dioxide and other “waste products” from the muscles.

During the development of children, their cardiopulmonary system will grow. This is the result of growth and maturation, but physical activity levels and disease factors can also have a significant impact on the cardiopulmonary system of youth. Especially during puberty, a rapid growth spurt in height and weight can be observed.

With growth of body height, especially of the thorax, the absolute size of the lungs and heart will also increase.

### Cardiac output

The increased size of heart and lungs will result in a changed in cardiopulmonary response to exercise. Heart size determines the stroke volume of the heart (the amount of blood pumped by the heart per heartbeat). This is an important determinant of the cardiac output:

Cardiac output (CO; L∙min ^-1^ )=stroke volume (SV; L)× heart rate (HR; beats∙min ^-1^ )(4).

### Minute ventilation

Another factor for the cardiopulmonary response to exercise is the minute ventilation (VE):

VE (L∙min ^-1^ )=tidal volume (TV; L)× breathing frequency (BF; breaths∙minute ^-1^ ). (5)

With the increase in height, an increase tidal volume with a concomitant decrease in breathing frequency is observed. The increase in tidal volume is very important to lower the relatively high anatomical dead space ventilation observed in children. Furthermore, it enhances the ability to increase VE to very high values (150–200 L∙min ^-1^ ) that can be observed in post-adolescent endurance athletes.

The decrease in breathing frequency during submaximal exercise as well as at maximal exercise intensity is of interest in children as this helps decrease the energy cost of breathing. Breathing frequency during exercise is determined by the mechanoreflex, metaboreflex, as well as the central command. Together with the tidal volume, breathing frequency drives the required VE during exercise.

It seems consensual in pediatrics that during incremental exercise the VE/VCO _2_ ratio decreases progressively, and rises again only at the end of the exercise test. This relation may be used to characterize ventilatory response and exercise capacity. It has been argued that a high VE/VCO _2_ ratio or slope may be associated with a bad prognosis, as it is related to a diminished capacity of pulmonary perfusion and cardiac output. In children, this ratio decreases with growth and age 15 .

### Maximal (or peak) oxygen uptake

The maximal amount of oxygen taken up in the body (VO _2_ peak) is an important performance and health indicator. This parameter is also influenced by growth and development. The oxygen uptake (VO _2_ ; mL∙min ^-1^ ) is defined as:

VO _2_ (mL∙min ^-1^ )=CO (mL∙min ^-1^ )× (CaO _2_ −CvO _2_ ; ml∙100 ml ^-1^ ) (6)

where ‘(CaO _2_ − CvO _2_ )’ is the arteriovenous difference in oxygen content (mL), which is related to oxygen extraction by the exercising muscles and the ability of the lungs to bind oxygen to the blood.

As described above, the CO will increase by growth and development, improving the transport of oxygen to the exercising muscle. Furthermore, since muscle mass also increases with growth and development, the ability to utilize oxygen (lower CvO _2_ ) will be higher in older children and adults. In addition, the hemoglobin levels in the blood will also increase with age, further increasing the oxygen transport capacity of the blood.

### Gender differences

Girls are not just small boys. Girls and boys differ in their response to exercise, as well as in the development of the cardiorespiratory system with growth and development. Therefore, gender-specific reference values are required for most parameters that also take growth and development into account. In general, girls and boys do not differ significantly in performance up to the onset of puberty 3 . Girls generally start puberty about 1.5 years earlier than boys, although there are significant differences in the age of onset of puberty among children. During puberty, many physiologic developments take place as described above.

VO _2peak_ values increase in both genders up to the age of 18–20 years. However, at the age of 18 the average VO _2peak_ is about 25% lower in girls compared to boys 3 . Since Dutch girls are, on average, 14 cm shorter and 12 kg lighter than boys at the age of 18 16 , this difference will impact their exercise capacity and response to exercise. Also, a difference in body composition impacts the gender difference in exercise capacity. Since females have a relatively higher fat mass for body mass, the fat-free mass (muscle mass) is lower for a given body mass. Since muscle mass is an important determinant of performance, females will have a lower relative VO _2peak_ /kg and peak work rate (W _peak_ ) per kilo gram of body mass (W _peak_ /kg) 3 . It is therefore advised to index the exercise capacity also to the fat-free mass of a subject 17 .

### Reference values

Different sets of reference values are available for children and adolescents. These reference values are used for comparison of the obtained CPET data. However, there can be a large difference between the difference sets of reference values 18 . We advise using the same set of reference values in the same patient. A recent case study shows the difference of applying different sets of CPET reference values in the same patient 18 .

We have previously developed reference values for pediatric CPET data for healthy (Dutch) children 3 . Many clinicians are using these references values. One of the drawbacks for clinicians working outside of our country using these reference values is the fact that Dutch children are among the tallest children in the world. So, a 12-year-old Dutch girl might be significantly taller than, for example, a French peer. As we know that height (and also weight) significantly influence performance, we have reanalyzed this data 3 and plotted the data against height ( Fig. 1 ).

Choosing the best set of reference values for CPET is not an easy task for clinicians. Many reference value sets are already pre-programmed into the software of the CPET system. However, it is not always clear how they are derived and whether they are applicable for children and adolescents. It is important to note that treadmill ergometry and cycle ergometry have specific reference values and these are not interchangeable. Further, adult reference values are not applicable for children and adolescents.

**Fig. 2:**
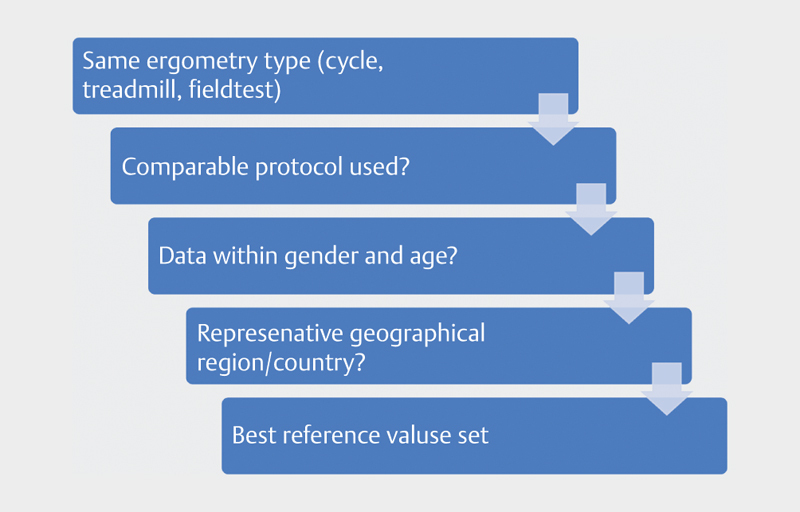
Flowchart for the selection of CPET reference values.

We have developed a flowchart for the selection of CPET reference values (see Fig. 2 ). First, it is important to look for reference values that have been obtained in in the same exercise mode (running/cycling). Then, it is important to check whether a comparable protocol has been used (maximal vs. sub-maximal exercise, RAMP vs. step, exercise duration). Due to gender differences and age effects, reference values should have been obtained in the same gender and age range. The next step is to check whether data from the same geographical region are available. Although there is not much research available on this topic, we know that there are racial differences in exercise physiology. Furthermore, there are differences noted between geographical regions. Values obtained in North America or Asia might not be valid to use in West-European subjects.

### CPET Interpretation and CPET Report

**Fig. 3:**
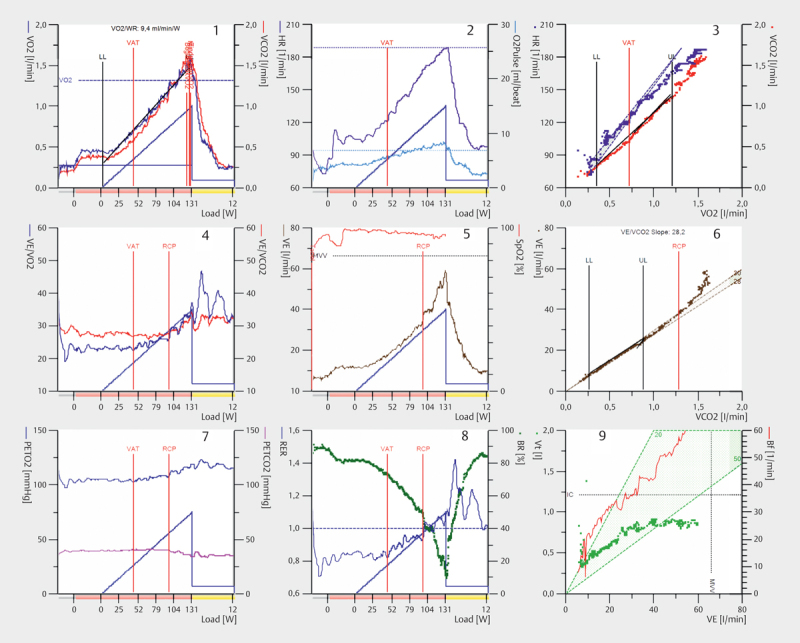
Example of a Wasserman 9-panel plot (2012 edition 30 ) for a healthy girl. For explanations and abbreviations, see text.

After the CPET has been completed, the test results need to be analyzed and a thorough interpretation should be made. Optimal utilization of CPET data requires valid and reliable collection and presentation of the data in a clear and standardized format. Several approaches are employed to graphically display the data, of which the 9-panel plots originally developed by Dr. Karl Wasserman MD PhD is the most popular approach 19 . In the following part, we will describe the parameters displayed in the 9-panel plot (see Fig. 3 for an example of a healthy child).

### Important parameters displayed in the 9-panel plot

#### Respiratory Exchange Ratio (RER; panel 8)

RER is calculated as the VCO _2_ divided by VO _2_ . RER reflects the substrate metabolism during CPET. It is usually around 0.8 at the start of the test and increases above 1.0 at maximal exercise. When RER is around 0.7 the subject is using mainly fatty acids as fuel, when the amount of glycogen/glucose as a fuel increases during exercise, the RER increases. When the RER>1.0, the body is mainly using glycogen/glucose as a fuel. The RER>1.0 is regarded as a criterion for maximal effort during CPET. In the recovery phase, the RER shows an overshoot, which is related to aerobic fitness 20 .

#### Heart rate (HR; panel 2)

HR is the number of heart beats per minute assessed using an ECG-system or heart rate monitor. Heart rate increases linearly with increasing intensity during CPET. The maximal HR (>95% of predicted) is used for identifying whether a subject is giving a maximal effort.

#### Minute ventilation (VE; panel 5)

VE increases almost linearly with exercise intensity during the first part of the CPET up to the first ventilatory anaerobic threshold (VT1 or VAT). After this point, a non-linear increase VE is observed. The maximal voluntary ventilation (MVV) is a surrogate of the maximum sustainable ventilatory capacity, a VE _peak_ /MVV ratio

<0.8–0.85 (i. e. breathing reserve>20–15%) is used to exclude ventilatory limitation to exercise. MVV of a subject can be estimated from the Forced Expiratory Volume in 1 second (FEV _1_ ) from a pulmonary function test (we advise doing this before every CPET): MVV=FEV _1_ x 35.

Breathing reserve above the threshold for abnormality (e. g. 20–30%) might

be relevant for the subject’s exertional dyspnea, if reached precociously during incremental exercise.

VE is related to the partial pressure of carbon dioxide in the arterial blood (PaCO _2_ ). An increase in PaCO _2_ triggers an increase in VE to maintain a relatively constant level of PaCO _2_ .

#### VE/VCO _2_ slope (panel 6)

A slope between the VE and VCO _2_ can be calculated, the VE/VCO _2_ slope. This slope is calculated using datapoints from the start of exercise up to the second ventilatory anaerobic threshold (VT2). Above VT2, there is a further increase in the VE/VCO _2_ slope. This part of the slope is not usually used to calculate the VE/VCO _2_ slope.

#### Oxygen pulse ( O _2_ pulse;=VO _2_ /HR; panel 2)

O _2_ pulse is an index of cardiac stroke volume. The O _2_ pulse should increase during exercise and often shows a plateau from moderate-intensity exercise. The predicted value for the peak O _2_ pulse is the predicted VO _2peak_ divided by the predicted HR _peak_ . A sudden decrease in the O _2_ pulse during exercise is a sign of a decreasing stroke volume and might indicate myocardial ischemia. However, the occurrence of myocardial ischemia is very rare in children and adolescents.

#### Oxygen uptake (VO _2_ ; panel 1)

VO _2_ increases linearly with workload during CPET. A normal increase is about 10 ml O _2_ per Watt. A plateau in VO _2_ in the final stages of a CPET is infrequently observed in children and therefore is not a very practical indicator of maximal effort. VO _2peak_ is the primary outcome of the CPET and the first performance indicator to look at. Also, the maximal workload (and the workload/kg) are important performance indicators (Panel 1).

#### Carbon dioxide exhalation VCO _2_ (panel 1)

VCO _2_ (panel 1) also increases linearly with workload in the first part of CPET. However, VCO _2_ shows a breakpoint after which it increases faster than the increase in VO _2_ . In the final stage of the CPET, VCO _2_ is higher than VO _2_ , and hence the RER (=VCO _2_ /VO _2_ ) is higher than 1.0.

#### Ventilatory equivalents for O _2_ and CO _2_ (panel 4)

The ventilatory equivalents for VO _2_ and VCO _2_ are the values expressed in relation to VE (VE/VO _2_ & VE/VCO _2_ ). These are called the ventilatory equivalents for oxygen and carbon dioxide. These parameters provide an indication of the ventilatory efficiency of a subject, a higher value indicating lower efficiency. VE/VO _2_ decreases during a CPET until the VT _1_ , after this point, VE/VO _2_ increases with incremental exercise intensity. VE/VCO _2_ decreases during CPET from the start to the VT _2_ . After this point, VE/VCO _2_ increases with incremental exercise intensity.

#### Tidal volume (TV) and breathing frequency (BF, panel 9)

As described above, VE is set by TV and BF. During CPET, VE increases in the initial stage of CPET by augmenting TV, during later stages of the CPET, VE mainly increases by an increase in BF. During exercise tidal volume/inspiratory capacity≥0.7, and a tidal volume plateau reached at an abnormally-low work rate 21 .

End-tidal carbon dioxide partial pressure (PETCO _2_ ) and end-tidal oxygen partial pressure (PETO _2_ ; panel 7)

PETCO _2_ and PETO _2_ remain quite stable (or show a small decrease in PETO _2_ , and a small increase in PETCO _2_ during sub-maximal exercise) during the first part of the CPET. The increase in PETO _2_ is an indicator of the VT _1_ , while the decrease in PETCO _2_ is the indicator of the VT _2_ . The lower limit of normal for PETCO _2_ is around 35 mm Hg.

#### Blood oxygen saturation (SpO _2_ %; panel 7)

SpO _2_ % can be monitored non-invasively during exercise at the finger, earlobe of forehead, and should stay around 100% and not decrease more than 4% from its baseline value. It is important to check the signal quality regularly during exercise since artifacts are quite common when monitoring the SpO _2_ % during exercise.

#### Blood pressure

Measuring blood pressure is also recommended during CPET. Systolic blood pressure (SBP) should increase depending on the workload of the subject 22 , while diastolic should remain stable during the test. A recent study from Sweden suggests an increase in SBP of 36 mmHg per 100 Watt of workload in children 23 .

#### Ventilatory thresholds (panel 3, 4, 6, 7)

The first ventilatory anaerobic threshold (VT _1_ or VAT) and second ventilatory threshold (VT _2_ or Respiratory Compensation Point (RCP) can be observed in panels 3, 4, 7 and 4, 6, 7 respectively. The VT _1_ is a submaximal index of aerobic fitness and can be identified as the intensity of the exercise where ventilation begins to increase in relation the oxygen uptake. Usually, the intensity of the VT _1_ is between 40–60 percent of the VO _2peak_ . A higher VT _1_ allows for a higher exercise intensity than a subject can sustain during endurance exercise.

In panel 3, VO _2_ is plotted against VCO _2_ . The point where the slope of the VO _2_ -VCO _2_ graph exceeds 1 demarcates VT _1_ . This method is called the V-slope method 24 .

VT _1_ can also be determined using a plot of VE/VO _2_ over time (panel 4), or PETO _2_ over time (panel 7). The VT _1_ is at the point where VE/VO _2_ and PETO _2_ start increasing from their nadir values. The VT _1_ is expressed as the VO _2_ value at the time point of VE/VO _2_ or PETO _2_ increase related to the predicted VO _2peak_ of the subject. For children and adolescents with an above-average VO _2peak_ , we advise using their own VO _2peak_ instead of the predicted VO _2peak_ .

The VT _2_ is recognized as the point during CPET at which an exponential increase in VE relative to VCO _2_ exhalation occurs 25 . At this intensity, the subject is no longer able to speak. Normally, the VT _2_ is between 60–90% of a subject’s VO _2peak_ , and a higher number indicates a better ability to perform in higher intensity exercise.

VT _2_ can be determined as the point where the linearity between VE and VCO _2_ cannot be maintained (panel 6). Above the VT _2_ there is an additional increase in VE for every increase in VCO _2_ . This point is also visible in panel 4, the VT _2_ is demarcated at the point where an increase in the VE/VCO _2_ ratio during exercise occurs. In panel 7, this point can be identified as the point where there occurs a concurrent decrease in PETCO _2_ . The intensity on the VT _1_ and VT _2_ can be used for a personalized exercise prescription following the polarized training approach 26 .

### CPET interpretation strategy

Previously, we described a 7-step CPET interpretation strategy for use in pediatric patients 27 . The reader is referred to the publication of van Brussel et al. 27 for more detailed information. We advise clinicians to use this strategy for the interpretation of CPETs. In short, the seven steps are:

1. The rationale for the CPET;
2. Check the data for technical errors. Especially the resting VO _2_ , VE and RER. If these parameters are physiologically too low (<0.25 l/min,<9 L/min or<0.7 respectively), this can be a sign for malfunctioning of the equipment or face mask leaks.
3. Rate the quality of the delivered effort. Is the HR _peak_ above 95% of predicted, or RER _peak_ above 1.0 (lower limits of normal)? Lower values might indicate a submaximal effort. A submaximal effort limits the interpretability of a CPET.
4. Determining aerobic fitness. Examine whether the VO _2peak_ and the VO _2peak_ /kg values are above -2 SD. Also, an abnormal body composition might result in a mismatch between VO _2peak_ and VO _2peak_ /kg 28 . There is much debate in the pediatric exercise physiology literature on how to normalize the VO _2peak_ data for body size and composition 29 . Normalization per kilogram of fat-free mass might be a good alternative.
5. Describe the physiological responses to exercise:
  - Describe the responses of the cardiovascular system and O _2_ transport;
  - Describe the responses of the respiratory system;
  - Describe the gas exchange and ventilation-perfusion matching;
  - Describe the muscle metabolism during exercise;
  - Are there signs of deconditioning (High HR during exercise, low VO _2_ peak, low VT _1_ [<40% of predicted VO _2peak_ ])?
6. Describe the dominant limiting factor for exercise. What is the reason for the child to terminate the CPET, and what is the physiological limitation based on the results of the CPET?
7. How was the effort perceived by the child? A Rating of Perceived Exertion (RPE) scale can help for this purpose.
8. Perform a clinical interpretation and generate a CPET report.

Several vendors have made efforts to develop standardized CPET reports. However, these reports are mostly focused on adult populations. Pediatric-specific issues such as a lower RER _peak_ and higher HR _peak_ during exercise, as well as different reference values for many physiologic parameters, are often not addressed in those reports.

In our experience with clinicians referring children for a CPET, the most frequently asked question is whether the child has a normal exercise response and a normal fitness level.

Also, clinicians want to know whether specific pathophysiological patterns/responses can be observed and whether their clinical question(s) can be answered (e. g. heart rhythm abnormalities or bronchoconstriction).

Although not all CPET parameters make sense to clinicians, a summary with a description the main CPET parameters should be provided. Furthermore, a standardized interpretation and conclusion should be given, preferably using the seven steps described above.

The CPET report should clearly state the “dominant” physiological limitation of the CPET, or whether the test was a symptom limited (sub-maximally performed) test. Finally, the report should provide a clear answer to the clinical question of the referring clinician, as this will help the referrer in the care or follow-up of the patient. Also, advice regarding sport and physical activity participation and body composition might be given.

## Conclusions

CPET can be performed in children for establishing a baseline biometric for which improved outcomes are serially tracked and ideally rewarded, as in most cases the fitness values of children with a chronic medical condition are very low.

CPET is best interpreted in light of the pre-test likelihood of abnormality, as well as the exercise-related complaints or symptoms of patients. In this article, we have described approaches to performing CPET in children, selecting reference values, and interpreting the CPET data.
